# Nonlinear and intensity-dependent enhancement of sweetness perception as a function of redness: Behavioral and EEG evidence for visual–gustatory cross-modal integration

**DOI:** 10.1016/j.fochx.2026.104075

**Published:** 2026-06-06

**Authors:** Yifei Sun, Yihan Wang, Yang Li, Feijie Xia, Tieyu Qian, Yangyang Wei

**Affiliations:** a Architecture and Design College, Nanchang University, Nanchang, China; b School of Creative Design, Wuhan Business University, Wuhan, China

**Keywords:** Sweetness perception, Redness manipulation, Cross-modal interaction, Sensory evaluation, EEG

## Abstract

Reducing sugar while maintaining perceived sweetness is a major challenge in beverage reformulation. This study systematically quantified the relationship between red color intensity and sweetness perception and evaluated its potential as a sensory compensation strategy. Forty participants assessed sucrose solutions (2.5–10.0% *w*/*v*) combined with graded redness levels under controlled conditions. Behavioral results demonstrated a nonlinear inverted U-shaped enhancement effect, with medium redness producing the greatest sweetness increase, particularly under low-sweetness conditions (maximum relative increase: 26.48%). The enhancement effect decreased as baseline sweetness increased, indicating clear intensity dependency. Objective neural measurements provided correlative evidence supporting the perceptual findings, reflecting the cognitive evaluation of the observed modulation effect. These results establish a quantifiable dose–response relationship between redness and sweetness perception and identify an optimal redness range for sweetness compensation. The findings provide practical guidance for color-based strategies in sugar-reduced beverage formulation.

## Highlights

• Redness enhances sweetness perception in a nonlinear inverted-U pattern.
• Medium redness (a* ≈ +20) yields optimal sweetness enhancement.
• Enhancement is intensity-dependent and strongest at low sweetness.
• Parietal LPC correlates with subjective sweetness ratings.
• EEG reveals multi-stage neural dynamics of cross-modal integration.

## Graphical abstract

**Figure:**
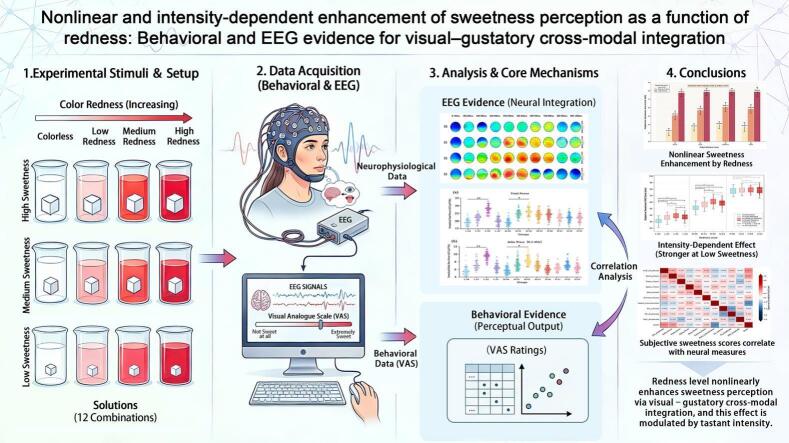

## 1 Introduction

Human perception of food flavor is a complex multisensory experience that depends not only on taste and olfaction but is also strongly influenced by the integration of visual, auditory, and tactile sensory information (Spence, 2015). Among these cues, color, as the earliest and most direct visual signal, plays a crucial role in shaping both taste expectations and actual perceptual experiences (Motoki, Spence and Velasco, 2023). A large body of research in food science and psychology has demonstrated robust cross-modal associations between color and taste (Chen et al., 2025; Spence, 2011). Cross-modal associations refer to the tendency for individuals, often without conscious awareness, to automatically match information from one sensory modality (e.g., vision) with information from another modality (e.g., taste), thereby enhancing the overall perceptual experience (Stein, Stanford and Rowland, 2014). Such associations typically operate implicitly, without reliance on deliberate intention or conscious awareness, and constitute one of the core mechanisms underlying multisensory integration research (Spence, 2018). Through shaping expectations of palatability, these mechanisms ultimately influence judgments of food acceptability and consumer decision-making (Dovey et al., 2012; Koch & Koch, 2003).

In the field of food sensory science, cross-modal associations between vision and taste have been extensively investigated. For example, when white wine is colored red or rosé, consumers tend to describe it using more descriptors typically associated with red wine compared with the uncolored version (Wadhera et al., 2014; Wang & Spence, 2020). Building on this principle, color cues have been widely applied in food packaging and labeling design to guide consumers' expectations of flavor attributes. For instance, cider presented with red labels has been perceived as sweeter and fruitier in sensory evaluations (Sugrue & Dando, 2018), and wines with red labels are more likely to elicit associations related to “fruitiness” (Lick et al., 2017). In addition, across a range of products such as breakfast cereals, ice cream, iced tea, and yogurt, red packaging has been consistently shown to effectively enhance consumers' perceived sweetness (Huang & Lu, 2015). These cross-modal associations are not formed arbitrarily, but rather emerge from long-term life experience and cultural learning, which render color a powerful external cue capable of pre-setting taste expectations (Baptista et al., 2021). In particular, red is commonly associated with sweetness and ripeness (Johnson & Clydesdale, 1982; Maga, 1974), whereas green is often linked to sourness or immaturity (Alley & Alley, 1998; Saluja & Stevenson, 2018). Such implicit and subconscious modulation of flavor perception by color is of particular research and practical significance in the current global context of sugar reduction and sugar control aimed at promoting healthier dietary patterns (Chonpracha et al., 2019).

Humans have an innate preference for sweetness (Holgerson et al., 2023; Liu & Bohórquez, 2022). In recent years, with growing public health awareness and the continuous global rise in the prevalence of metabolic diseases such as obesity and diabetes, reducing the intake of “added sugars” in the diet has become an urgent public health priority (Anjana et al., 2025; Fałczyńska et al., 2025; Peñalvo, 2024). Excessive consumption of sugar-containing foods, particularly sugar-sweetened beverages (SSBs), has been shown to be significantly associated with multiple health risks, including obesity (Fulton et al., 2022; Malik & Hu, 2022), type 2 diabetes (Masood & Moorthy, 2023; Moores et al., 2022), and cardiovascular diseases (Debras et al., 2020; Gao et al., 2021). To address this challenge, the World Health Organization (WHO) and governments in many countries have issued policy recommendations suggesting that daily energy intake from added sugars should be limited to less than 10%, with a further reduction to 5% to achieve additional health benefits (World Health Organization , 2015). In parallel, multiple fiscal policies, such as the sugar tax implemented in the United Kingdom, have been enacted (Rogers et al., 2023) with the aim of reducing SSB consumption through economic incentives (Lou et al., 2021; Moore et al., 2023).

Against this backdrop, sugar reduction has become an irreversible development trend in the beverage industry (Chen et al., 2022). However, sugar contributes not only to sweetness but also plays a critical role in food texture, flavor release, and shelf-life stability (Erickson & Carr, 2020). Direct sugar reduction often leads to a significant decline in product acceptability (Di Monaco et al., 2018). Therefore, the development of effective sugar-reduction strategies is essential. Current mainstream approaches include direct sugar reduction, the use of sweeteners (De Souza et al., 2021; Zhu et al., 2021), and sweetness enhancers (Karl et al., 2020). In addition, enzymatic sugar reduction, heterogeneous sugar distribution, and cross-modal effects have emerged as novel strategies for sweetness enhancement under reduced-sugar conditions. Enzymatic sugar reduction replaces part of the added sugar by hydrolyzing lactose into sweeter glucose (Wahab et al., 2021), although its application remains limited. Heterogeneous sugar distribution can enhance sweetness perception through asynchronous stimulation of sweet taste receptors (STR) (Khemacheevakul et al., 2021); however, this approach is mainly applicable to solid or semi-solid foods and is difficult to implement in beverage systems due to matrix constraints. Among these strategies, multi-sensory integration demonstrates unique advantages and considerable potential. By systematically leveraging interactions among different sensory modalities, such as vision and olfaction, this approach can enhance perceived sweetness without altering actual sugar content (Chen et al., 2020; Hu et al., 2025), thereby offering a highly promising solution for achieving “sugar reduction without sweetness reduction” in beverage formulation design (Oliveira et al., 2021; Tan & Pang, 2025).

Color modulation, as a low-cost and easily implementable approach, is considered to possess high industrial feasibility. A substantial body of research has demonstrated that specific colors—particularly red—can significantly enhance consumers' perceived sweetness intensity (Gous et al., 2019; Spence et al., 2015), potentially through the induction of experience-based taste expectations or via direct cross-modal integration (Spence, 2024). Mechanistically, the modulatory effect of color on taste perception is often conceptualized as “sensation transference,” whereby affective responses elicited by one sensory cue (e.g., color) are transferred and systematically influence the evaluation of other sensory attributes (e.g., taste) (Spence, 2022). Specifically, the influence of color on taste perception involves two distinct levels of transformation: the first is expectation-based cognitive modulation, in which flavor expectations evoked by color (e.g., red signaling greater sweetness) exert top-down influences on subsequent gustatory processing (Spence et al., 2010); the second is rapid and automatic cross-modal integration, whereby visual information is integrated with gustatory input at early sensory cortical stages, directly modulating the neural response strength of taste signals. Importantly, the regulatory effect of color on taste perception is not solely attributable to psychological association but is supported by a neurophysiological basis. Sweetness perception itself is a complex process involving peripheral receptor activation and central nervous system integration (Roper, 2013). However, the sweetness-enhancing effect of color does not occur uniformly across contexts, and its magnitude may be modulated by baseline sweetness levels. Without systematic quantification of the relationship between color intensity and perceptual outcomes, the application of color-based strategies in reduced-sugar products may remain largely empirical.

On the other hand, methodological limitations in existing empirical research have hindered practical exploration and precise quantification of the underlying mechanisms. Most studies continue to rely primarily on behavioral subjective sensory evaluations, which are characterized by strong subjectivity and high inter-individual variability (Barajas-Ramírez et al., 2024; Shariati et al., 2025), thereby reducing reproducibility and limiting the reliability of the conclusions drawn. Moreover, traditional behavioral approaches are insufficient to capture the dynamic and complex neural processes involved in color-modulated sweetness perception, constraining mechanistic interpretation. In machine perception research (e.g., electronic tongue systems), limitations in sensor type and number restrict adaptability to diverse novel product matrices (Mahanti et al., 2024). Therefore, introducing objective methodologies capable of directly reflecting sensory modulation effects is essential to enhance the reliability and reproducibility of sensory evaluation. Electroencephalography (EEG), with its millisecond-level temporal resolution, provides an ideal tool for investigating such rapid cognitive processes (Domracheva & Kulikova, 2020; Mheich et al., 2021). EEG not only enables direct measurement of brain responses to sensory stimuli and yields objective, quantifiable neural data, but also facilitates the capture of real-time neural dynamics associated with sweetness perception, thereby revealing temporal patterns of brain activity beyond the reach of traditional sensory assessment methods (Loosen et al., 2025). By analyzing event-related potentials (ERPs) and neural oscillations (e.g., alpha and gamma bands) in the time–frequency domain, EEG can delineate the temporal sequence and mechanisms of information processing during cross-modal integration, providing more reliable and reproducible neural evidence (Bauer, Debener and Nobre, 2020). In addition, most existing studies are based on binary comparisons of color presence/absence or high/low chromatic intensity, lacking systematic testing across graded visual color levels, which limits precise quantification of dose effects in food formulation design. Although some studies have examined the influence of color congruency (Peng, Sun and Wan, 2022), the dose–response relationship and exact perceptual thresholds between physical color attributes and sweetness enhancement remain unclear.

In summary, the present study focuses on the color–taste interaction within multisensory integration and aims to systematically investigate the cross-modal enhancement effect of red visual cues on sweetness perception and its underlying neural mechanisms using high-temporal-resolution EEG technology. Specifically, the study seeks to behaviorally verify the sweetness-enhancing effect of red visual signals across different baseline sweetness levels, identify specific EEG neural markers associated with this enhancement, and examine their consistency with subjective ratings. Furthermore, the study aims to establish the functional relationship between red visual intensity and perceived sweetness intensity and to determine the corresponding enhancement threshold. Based on these objectives, the following hypotheses were proposed:

H1: Compared with colorless solutions, sucrose solutions of identical sweetness supplemented with a carminic acid–based colorant will be perceived as significantly sweeter;

H2: The magnitude of sweetness enhancement will exhibit a nonlinear relationship with red visual intensity, with a measurable redness threshold;

H3: The sweetness enhancement effect of red solutions will be accompanied by specific EEG changes, and these neural indices will be significantly correlated with subjective sweetness intensity ratings.

By quantifying the neural mechanisms underlying color–taste cross-modal interaction, this study provides preliminary and practically applicable scientific evidence to support sugar-reduction strategies in the food industry. Through systematic elucidation of the compensatory effect of color on sweetness perception, the findings further inform the development of optimized color compensation strategies in food processing. Such strategies may effectively compensate for sweetness loss and enhance consumer acceptance while avoiding potential health risks associated with excessive colorant use. The study thus provides both theoretical support for the application of color-based flavor modulation in low-sugar foods and scientific guidance for practical implementation in the food processing industry.

## 2 Materials and methods

### 2.1 Participant recruitment

A total of 40 right-handed adult participants (20 males and 20 females) were recruited for this study, aged between 18 and 35 years (M = 23.6, SD = 3.1). The sample size was determined using an a priori statistical power analysis conducted with G*Power 3.1 software (Bartlett et al., 2022). Assuming an effect size of f = 0.25, a significance level (α) of 0.05, and a statistical power (1 − β) of 0.90, the analysis indicated a minimum required sample size of 22 participants; therefore, 40 participants were ultimately recruited to adequately account for potential data loss and to ensure the robustness of the results. All participants reported being in good health and right-handed (Sha et al., 2021), with no history of psychiatric or neurological disorders, and no impairments in olfactory, gustatory, or visual function (including no color blindness or color vision deficiency, as screened using the Ishihara color vision test). Participants also reported no history of food colorant allergies or metabolic diseases. In addition, all participants were non-smokers, and pregnant or breastfeeding women were excluded. During the week prior to the experiment, participants were instructed to avoid taking any medications that could affect taste perception or central nervous system function. After receiving a full explanation of the experimental procedures, each participant provided written informed consent and was informed of their right to withdraw from the study at any time without penalty. The study protocol was approved by the Biomedical Research Ethics Committee of the Second Affiliated Hospital of Nanchang University (Approval No.: I-Yiyan Lunshen [2025] No. (138)), and participants received appropriate compensation upon completion of the experiment.

### 2.2 Experimental materials and solution preparation

#### 2.2.1 Preparation of baseline solutions

Reverse osmosis–treated purified water was used as a tasteless carrier to minimize potential interference of water quality on sweetness perception. Food-grade colorless sucrose (Sigma-Aldrich, purity ≥99.5%) was used to prepare three baseline solutions with different sweetness levels:

a) Low sweetness (L): 2.5% *w*/*v* (100 mL water +2.5 g sucrose).

b) Medium sweetness (M): 5.0% w/v (100 mL water +5.0 g sucrose).

c) High sweetness (H): 10.0% w/v (100 mL water +10.0 g sucrose).

These concentration gradients were determined based on pilot testing to produce clearly distinguishable sweetness levels without inducing excessive sensory adaptation or aversion, and to correspond approximately to 20%, 50%, and 80% of the perceived sweetness range on the visual analog scale (VAS).

#### 2.2.2 Preparation of colored solutions

First, a tasteless and odorless food-grade water-soluble red colorant (carminic acid, E120) was used to manipulate the color variable. The a* coordinate in the CIE L*a*b* color space was adopted as the primary objective quantification index. Red visual intensity was controlled by diluting a concentrated stock colorant solution. The CIE L*a*b* chromaticity values were measured under a standard D65 illuminant using a spectrophotometer (Konica Minolta CM-5), and chroma $C_{\mathrm{ab}}^{*}$ and hue angle (h*) were calculated as follows:

$$C_{\mathrm{ab}}^{*}=\sqrt{{a^{*}}^{2}+{b^{*}}^{2}}$$

Based on pilot testing, four redness levels were established:

a) S0 (0%): colorless control, a* ≈ −1.0.

b) S1 (low redness): 0.003% carminic acid colorant, target a* ≈ +10.0.

c) S2 (medium redness): 0.01% carminic acid colorant, target a* ≈ +20.0.

d) S3 (high redness): 0.05% carminic acid colorant, target a* ≈ +35.0.

The concentration range of carminic acid selected in this study (0.003%–0.05% *w*/*v*) was based on commonly used addition levels in the food industry, and the highest addition level (S3) also strictly complied with the maximum permitted limit for food colorants in beverages specified in China's GB 2760 standard, thereby ensuring the safety and practical feasibility of the sensory compensation strategy within the regulatory framework. The mean ± standard deviation values of L*, a*, b*, $C_{\mathrm{ab}}^{*}$, and h* for each sample are provided in the Supplementary Materials (Table S1). Measurements indicated that as redness level increased, both a* and $C_{\mathrm{ab}}^{*}$ showed a monotonic upward trend, whereas changes in b* were minimal, indicating that color manipulation was primarily conducted along the redness axis without introducing significant hue shifts.

To ensure consistency in baseline gustatory properties across different redness levels, the highest redness solution (S3) was prepared first, and the remaining redness levels (S1 and S2) were subsequently obtained by quantitatively diluting S3 with the corresponding colorless sucrose base solution of identical concentration. To exclude potential olfactory interference, all solutions were analyzed using gas chromatography–mass spectrometry (GC–MS) to confirm the absence of interfering volatile compounds, thereby ensuring no significant differences in gustatory or olfactory attributes other than red visual intensity.

Finally, the three baseline sweetness levels were fully crossed with the four redness levels, resulting in a total of 12 experimental stimulus solutions. All solutions were freshly prepared within 24 h prior to the experiment, aliquoted into 50 mL light-protected amber glass bottles, and stored at 4 °C. They were removed from refrigeration one hour before testing and allowed to equilibrate to room temperature (24 ± 1 °C) prior to use.

### 2.3 Experimental procedure

**Fig. 1:**
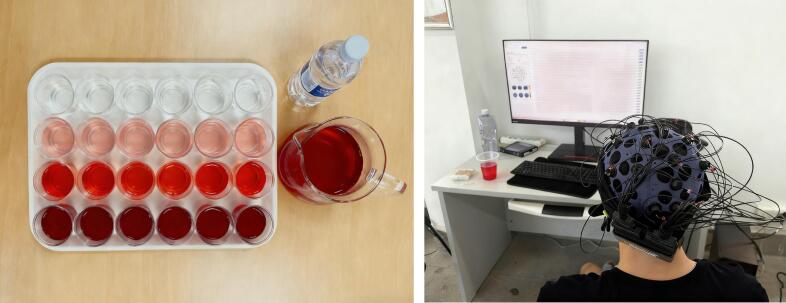
Experimental stimulus solutions and experimental setup.

The experiment was conducted in a quiet, odor-free, temperature-controlled (24 ± 2 °C), and light-controlled EEG laboratory (Fig. 1). Ambient illumination was provided by a standard D65 light source to eliminate chromatic deviation and to maintain consistent lighting conditions across all experimental sessions, thereby preventing interference from environmental light on color perception. Participants were instructed to abstain from eating and drinking (except water) for at least 2 h prior to the experiment and to wash their hair the day before testing to ensure optimal EEG signal quality.

**Fig. 2:**
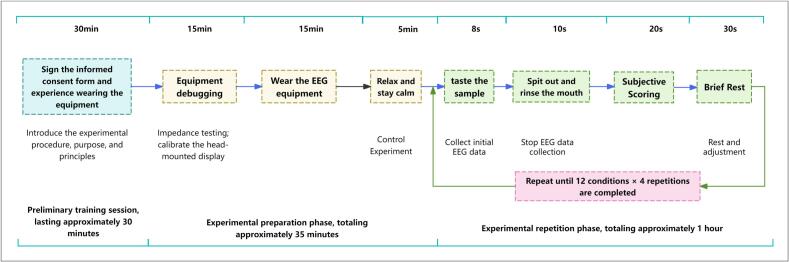
Experimental procedure.

The experiment adopted a within-subjects design. Each participant completed all 12 conditions (3 sweetness levels × 4 redness levels), with each condition repeated four times, resulting in a total of 48 trials. A Williams Latin Square design was used to counterbalance the presentation order of the 12 solutions, and sweetness levels were fully randomized within each block to eliminate order and fatigue effects (Richardson, 2018). Prior to the formal experiment, a 5-min resting-state EEG was recorded as baseline. Participants were instructed to sit comfortably with eyes closed and remain relaxed. The baseline data were used to normalize subsequent task-related EEG recordings to reduce inter-individual variability and background noise (Haegens et al., 2014). The procedure for a single trial was as follows (Fig. 2):

a) Preparation phase (2 s): A fixation cross (“+”) was presented at the center of the screen for 500 ms, accompanied by a voice prompt indicating the start of the trial.

b) Visual exposure (5 s): The experimenter presented a transparent glass containing approximately 10 mL of the stimulus solution (placed against a white background panel). Participants were instructed to visually inspect the solution for 5 s to ensure sufficient visual exposure.

c) Gustatory delivery (2 s): Participants used a straw to deliver approximately 5 mL of the solution to the central region of the tongue. The moment the solution contacted the tongue surface was marked as stimulus onset in the EEG recording system.

d) Taste retention and recording (5–8 s): Participants held the solution in their mouth (without rinsing or swallowing), fully experiencing the taste while maintaining head position and minimizing facial muscle movement to reduce motion artifacts. EEG signals were continuously recorded during this period.

e) Expectoration and rinsing (10 s): After fully tasting the solution, participants expelled it into a waste container and rinsed their mouth with purified water to remove residual taste and prevent interference from the preceding trial on subsequent trials.

f) Subjective rating (20 s): Participants rated the perceived “sweetness intensity” (anchored from “not sweet at all” to “extremely sweet”) using a 100 mm visual analog scale (VAS) presented on a computer screen, indicating their response by clicking the corresponding position with a mouse. The VAS provides high-resolution continuous data and is sensitive to subtle gradients in sweetness perception (Bailey et al., 2012), which is essential for quantifying dose–response relationships and determining perceptual thresholds. Moreover, it minimizes response bias associated with categorical scales and generates continuous variables that are highly compatible with EEG indices, providing an optimal basis for linking behavioral and neural data.

g) Inter-trial interval (30 s): Relaxing music was played during a 30-s rest period to allow recovery of taste receptors and prevent adaptation effects.

To further minimize gustatory adaptation and fatigue, the experiment was divided into four blocks, each containing 12 trials. A mandatory rest period (≥5 min) was provided between blocks.

### 2.4 EEG signal acquisition and preprocessing

**Fig. 3:**
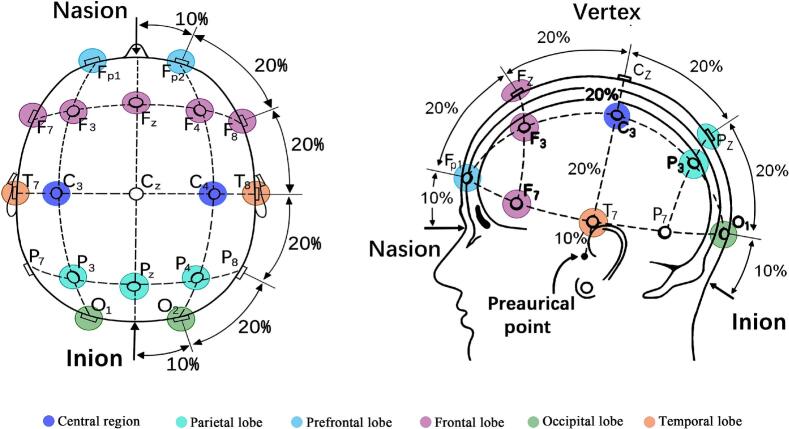
Electrode placement layout.

EEG data were acquired using a Bitbrain wearable 16-channel water-based electrode system, with electrodes positioned according to the international 10–20 system (Fig. 3). The sampling rate was set to 1000 Hz, and electrode impedance was maintained below 5 kΩ throughout the experiment. Horizontal and vertical electrooculogram (EOG) signals were simultaneously recorded to monitor ocular artifacts.

According to the experimental design, a total of 1920 EEG epochs could be collected. Raw EEG data were preprocessed in MATLAB 2023b (The MathWorks, United States of America) using the EEGLAB toolbox (Delorme & Makeig, 2004). The preprocessing pipeline first involved importing the raw data and electrode location information, removing irrelevant channels, and re-referencing the data to the average of the bilateral mastoids (M1 and M2). A 0.1 Hz high-pass filter was applied to the continuous data, and a 50 Hz notch filter was used to remove power-line interference, after which the sampling rate was uniformly downsampled to 500 Hz. EEG epochs of 1000 ms following gustatory stimulus onset were extracted based on event markers. Subsequently, individual bad channels with persistently poor signal quality were marked and temporarily removed to ensure full rank of the data matrix. To avoid the risk of artifacts caused by orofacial muscle activity during the taste-retention window, all participants received standardized training before EEG recording. During taste retention, participants were explicitly instructed to keep their head still, relax their facial muscles, keep the tongue flat, and refrain from swallowing, while the experimenter monitored the raw EEG signals in real time to mark obvious movement events.

After data decomposition using independent component analysis (ICA), components were automatically classified using the ICLabel tool. Independent components labeled as muscle activity (Muscle ≥80%), ocular activity (Eye ≥80%), or cardiac activity (Heart ≥80%) were marked as artifact candidates and were then independently inspected by two researchers with experience in EEG analysis (Zhao et al., 2024). Components with typical electromyographic (EMG) characteristics were specifically excluded: their topographical distributions were usually concentrated along the temporal margins near the orofacial muscles, such as T7, T8, F7, and F8, and their power spectra showed sustained broadband high-power distributions above 20 Hz. After thorough artifact cleaning, previously removed bad channels were reconstructed using spherical interpolation. Finally, at the trial level, epochs with peak-to-peak amplitudes exceeding ±100 μV were entirely rejected.

Previous studies have shown that excessively short time intervals may primarily reflect sensory adaptation and responses to stimulus novelty, whereas overly long intervals are more susceptible to noise contamination or attentional fluctuations (Dalton, 2000; Patterson et al., 2013; Wu et al., 2022). Considering the characteristics of the present experiment, a 0–1000 ms time window following the onset marker of gustatory delivery was ultimately selected for subsequent analysis, as neural responses to gustatory stimuli during this period are relatively stable and reliable (Katz et al., 2001).

The preprocessed EEG data were analyzed using custom MATLAB scripts in both the time and frequency domains. The analyses focused on variations associated with stimulus type (comparisons among different redness levels), brain region (regional activity differences), electrode channel (site-specific signal differences), and frequency band (power changes in δ, θ, α, β, and γ bands). Finally, correlation and regression analyses were conducted to examine the relationships between these neural indices and participants' subjective sweetness intensity ratings.

### 2.5 Data analysis methods

All statistical analyses of behavioral and EEG data were conducted using MATLAB 2023b and SPSS 27.0. Figures were generated using Python (3.9) with the Matplotlib (3.5.2) and Seaborn (0.11.2) libraries to ensure reproducibility and precision of graphical outputs.

For behavioral data analysis, subjective sweetness intensity ratings were averaged across the four repetitions for each participant under each condition and analyzed using repeated-measures analysis of variance (Repeated-Measures ANOVA). This analysis was used to examine the main effects of baseline sweetness and red visual signals, as well as their interaction. Bonferroni correction was applied for post hoc comparisons. When the assumption of sphericity was violated, Greenhouse–Geisser correction was used. If a significant interaction was observed, simple effects analyses were conducted. All post hoc comparisons were Bonferroni-corrected, and effect sizes were reported as partial η^2^.

EEG data analysis comprised both time-domain and frequency-domain approaches and was subsequently integrated with behavioral data.

Time-domain analysis focused on EEG activity within the 0–1000 ms time window following gustatory stimulus delivery. A large body of research has shown that the LPC is one of the most clearly characterized ERP components in cognitive neuroscience, with its maximum amplitude typically observed over the parietal midline and adjacent regions (Hajcak et al., 2010; Polich, 2007). This topographical distribution has been repeatedly validated across different sensory modalities (Cai et al., 2024; Schienle et al., 2025). Therefore, in the present study, the midline parietal electrode Pz and its neighboring electrodes P3 and P4 were preselected to constitute the region of interest (ROI). Event-related potentials (ERPs) were calculated for each condition, and the mean LPC amplitude in the key brain regions was extracted as the primary index. Repeated-measures ANOVA was used to examine differences in LPC amplitude under different red visual intensity conditions, and FDR (False Discovery Rate) correction was applied to control the risk of Type I errors arising from multiple comparisons.

Frequency-domain analysis aimed to further elucidate the neural oscillatory mechanisms underlying red visual modulation of sweetness perception. Power spectral density (PSD) was estimated using the Welch method based on short-time Fourier transform (STFT) and segment-averaging techniques. Specifically, the continuous signal was divided into overlapping segments, STFT was applied to each segment to compute PSD, and the resulting spectra were averaged to obtain a smoother and lower-variance frequency estimate. Analysis was conducted within the 0–1000 ms post-stimulus window. EEG data were decomposed into five classical frequency bands: delta (0.5–4 Hz), theta (4–8 Hz), alpha (8–13 Hz), beta (13–30 Hz), and gamma (30–45 Hz). Although gamma oscillations are closely associated with higher-order cognitive integration, they were included in the exploratory analysis in the present study because they are considered one of the indicators of cross-modal feature binding and conscious perception (Buzsáki & Wang, 2012; Meador et al., 2002). The area under the curve (AUC) of the PSD curve within the analysis time window was calculated for each frequency band and used as a quantitative measure of band-specific power.

Event-related spectral perturbation (ERSP) dynamic time–frequency analysis was performed on the preprocessed EEG data. Based on Morlet wavelet transform (wavelet number k = 5), EEG signals within the 0–1000 ms time window following the gustatory stimulus marker were subjected to time–frequency decomposition, with a frequency range of 0.5–45 Hz and a time step of 10 ms. The 5-min resting-state EEG recorded before the experiment was used as the reference baseline.

To examine the association between neural activity and behavioral perception, Spearman correlation analyses were further conducted between key EEG indices and subjective sweetness intensity ratings (VAS) across the 40 participants. The significance level for all statistical tests was set at α = 0.05.

## 3 Results

### 3.1 Sensory sweetness analysis results

To examine the cross-modal enhancement effect of red visual cues on sweetness perception, subjective sweetness intensity ratings (100 mm visual analog scale, VAS) from 40 participants were first analyzed under the 3 baseline sweetness levels (Low, L; Medium, M; High, H) × 4 redness levels (S0: colorless control; S1: low redness; S2: medium redness; S3: high redness) conditions. Descriptive statistics, repeated-measures analysis of variance (Repeated-Measures ANOVA), and Bonferroni-corrected post hoc tests were employed to systematically evaluate the main effects of color and sweetness, their interaction effects, and nonlinear relationships. The results were further analyzed in relation to the cognitive mechanisms underlying multisensory integration.

#### 3.1.1 Descriptive statistics

The mean subjective sweetness ratings, standard deviations (Mean ± SD), and increases relative to the colorless control (S0) under different conditions are presented in Table 1. The results indicate that the relative magnitude of the color-induced enhancement effect decreased as baseline sweetness increased (L > M > H).

**Table 1:** Subjective sweetness VAS ratings under different baseline sweetness and redness conditions (mm, *n* = 40).

Baseline sweetness	Redness level	Mean ± SD	Difference from S0 (mm)	Increase (%)
Low sweetness **L** (2.5% sucrose)	S0 (colorless)	31.27 ± 4.31	–	–
	S1 (low redness)	36.72 ± 4.36	5.46	17.46
	S2 (medium redness)	39.54 ± 4.06	8.28	26.48
	S3 (high redness)	37.26 ± 5.30	5.99	19.18
Medium sweetness **M** (5.0% sucrose)	S0 (colorless)	50.92 ± 5.32	–	–
	S1 (low redness)	56.97 ± 5.12	6.05	11.89
	S2 (medium redness)	60.79 ± 5.65	9.87	19.38
	S3 (high redness)	58.57 ± 5.11	7.65	15.02
High sweetness **H** (10.0% sucrose)	S0 (colorless)	77.63 ± 4.55	–	–
	S1 (low redness)	78.85 ± 4.46	1.22	1.58
	S2 (medium redness)	79.07 ± 4.01	1.44	1.86
	S3 (high redness)	78.92 ± 5.45	1.30	1.67

**Fig. 4:**
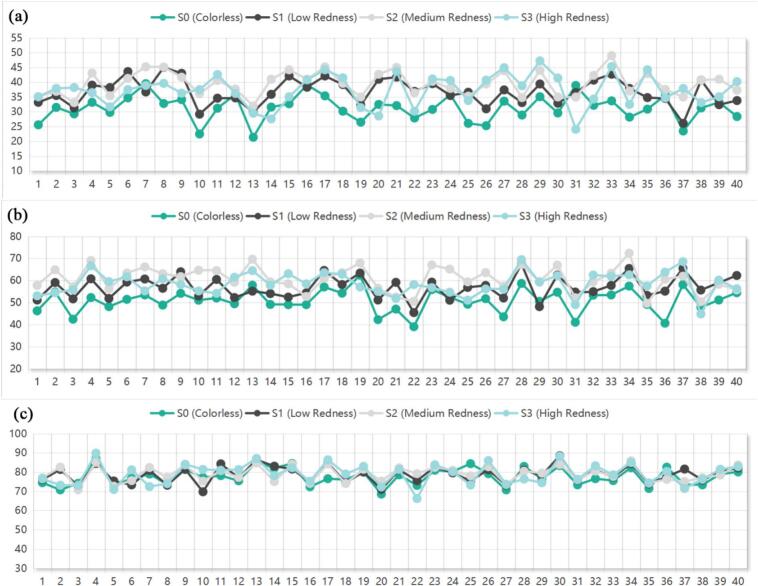
Subjective sweetness perception of solutions with different red visual intensities. Subjective sweetness visual analog scale (VAS) ratings for the four red visual intensity levels (S0: colorless control; S1: low redness; S2: medium redness; S3: high redness) under (a) low sweetness, (b) medium sweetness, and (c) high sweetness conditions (*n* = 40). (For interpretation of the references to color in this figure legend, the reader is referred to the web version of this article.)

**Fig. 5:**
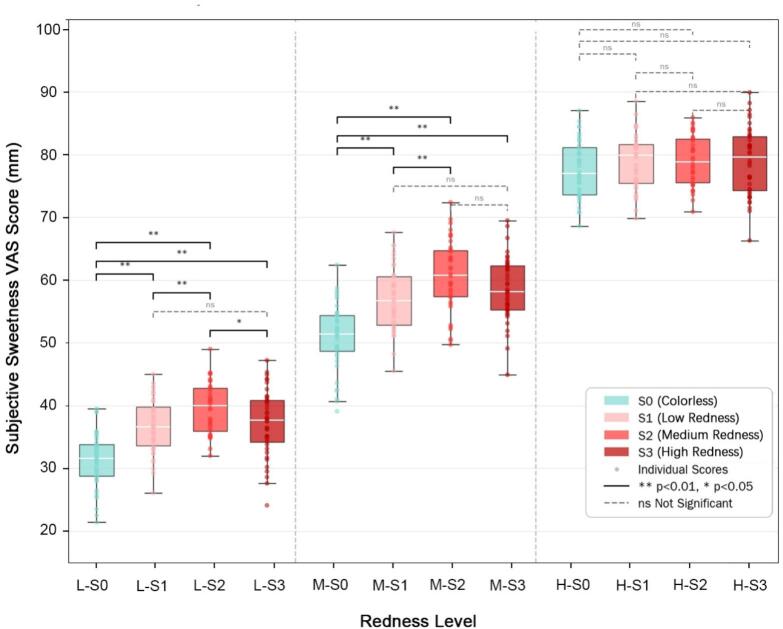
Boxplots of subjective sweetness ratings.

The line plots of subjective sweetness perception under different red visual intensity conditions (Fig. 4) illustrate both individual trajectories and overall trends in mean sensory sweetness ratings across all participants under each condition. The x-axis represents participant number (1–40), and the y-axis represents sweetness perception VAS scores (mm, range 0–100 mm). Overall, under low and medium sweetness conditions, the lines corresponding to S1, S2, and S3 generally lie above the S0 baseline. In contrast, under high sweetness conditions, the curves become flatter and largely overlap. Notable inter-individual differences were observed in slope, peak position, and magnitude of decline. For example, some participants exhibited a steep increase in the S2 condition, indicating heightened sensitivity to the enhancement effect of medium redness. Conversely, a small number of participants showed relatively flat trajectories or even crossovers among redness levels, suggesting weaker susceptibility to color influence or distinct cognitive evaluation strategies toward red visual cues. The boxplots (Fig. 5) further quantify differences in distribution dispersion. The boxes represent the interquartile range (IQR), the central line indicates the median, the whiskers denote 1.5 times the IQR, and the scatter points represent individual observations. The results show that under low and medium sweetness conditions, sweetness ratings followed an inverted U-shaped trend with increasing redness: ratings increased from S0 (colorless) to S2 (medium redness) and declined from S2 to S3 (high redness). Medium redness (S2) yielded the highest perceived sweetness, as reflected by the highest box position, median, and densest concentration of data points compared with other redness levels. This pattern was nearly absent under high sweetness conditions.

The above figures indicate that the magnitude of color-induced sweetness enhancement decreased markedly as baseline sweetness increased. Specifically, the low-sweetness group exhibited the largest increase from S0 to S2 (8.28 mm; relative increase 26.48%), followed by the medium-sweetness group (9.87 mm; relative increase 19.38%), whereas the enhancement effect in the high-sweetness group was nearly negligible (1.44 mm; relative increase 1.86%). In terms of inter-individual variability, the high-sweetness group under the high-redness condition (S3) showed the greatest dispersion in ratings (range: 66.3–89.9), the widest box (interquartile range = 8.62), and a standard deviation of 5.45, indicating substantial divergence in subjective perception under this condition. Although the absolute increase in sweetness due to color was largest at the medium sweetness level (S2 vs. S0: 9.87 mm), the relative enhancement rate was highest under low sweetness (26.48%), suggesting that visual cues exert a stronger gain effect when gustatory signals are weaker. These results systematically demonstrate that the modulatory effect of red visual intensity on sweetness perception is regulated by baseline sweetness level and that visual–gustatory cross-modal integration exhibits pronounced intensity dependency.

#### 3.1.2 Repeated-measures ANOVA results

**Fig. 6:**
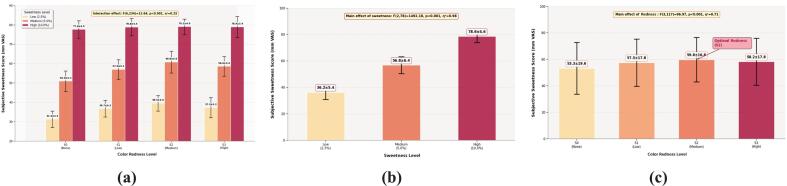
Effects of redness and baseline sweetness on subjective sweetness ratings. (a) Interaction between sweetness and redness; (b) main effect of baseline sweetness on subjective sweetness ratings; (c) main effect of redness on subjective sweetness ratings.

Because Mauchly's test of sphericity indicated that the data violated the assumption of sphericity (sweetness: W = 0.747, *p* = 0.004; redness: W = 0.683, *p* = 0.013; interaction: W = 0.373, *p* = 0.015), Greenhouse–Geisser-corrected results were used. The analysis showed (Fig. 6) a significant main effect of red visual intensity (F(3,117) = 96.97, *p* < 0.001, η^2^ = 0.71), a significant main effect of baseline sweetness (F(2,78) = 1492.18, p < 0.001, η^2^ = 0.98), and a significant interaction between the two factors (F(6,234) = 12.64, p < 0.001, η^2^ = 0.25). These findings indicate that red visual intensity exerts an overall modulatory effect on sweetness perception and that this effect is significantly influenced by baseline sweetness level. The relatively large partial η^2^ values observed in this study can be attributed, in part, to the repeated-measures design, in which inter-individual variability is removed from the error term, thereby increasing the proportion of explained variance relative to residual variance. At the same time, they objectively reflect the strong behavioral differences induced by the manipulated independent variables, particularly the stepwise changes in sucrose concentration. Together, these results further support Hypothesis H2 and demonstrate the high sensitivity of the experimental design to the target effects.

#### 3.1.3 Bonferroni post hoc comparisons and simple effects analysis

To further elucidate the significant interaction between sweetness and redness, post hoc tests were conducted for the main effects of sweetness and redness, respectively. Simple effects analyses were further performed to examine the specific patterns by which redness influenced sweetness perception under different baseline sweetness levels.

Bonferroni post hoc comparisons for the main effect of baseline sweetness (Table 2) indicated significant differences in perceived sweetness ratings among all three sweetness levels (all p < 0.001). Specifically, the mean rating for the medium-sweetness solution (56.812 ± 6.413) was significantly higher than that for the low-sweetness solution (36.198 ± 5.425), with a mean difference of 20.614. The high-sweetness solution (78.616 ± 4.639) was significantly higher than the medium-sweetness solution, with a mean difference of 21.804. The largest difference was observed between the high- and low-sweetness solutions, with a mean difference of 42.418. These results confirm that baseline sweetness is the primary determinant of perceived sweetness intensity.

**Table 2:** Bonferroni-corrected post hoc comparisons for the main effect of baseline sweetness.

Comparison	Mean difference (mm)	Standard error	*t* value	*p* value
Medium − Low	20.614	0.89	23.169	<0.001
High − Medium	21.804	0.551	39.599	<0.001
High − Low	42.418	0.845	50.185	<0.001

The overall analysis of the main effect of redness (Table 3) showed that, except for the comparison between S1 and S3 (mean difference = 0.735, *p* = 0.473), all other pairwise comparisons among redness levels reached statistical significance. Among them, the S2 (medium redness) condition yielded significantly higher sweetness ratings (59.800) than all other conditions, whereas the S0 (colorless) condition produced the lowest ratings (53.270). These findings preliminarily indicate that red visual intensity exerts an overall modulatory effect on sweetness perception, with S2 representing a potential optimal enhancement level.

**Table 3:** Bonferroni-corrected post hoc comparisons for the main effect of redness.

Comparison	Mean difference (mm)	Standard error	*t* value	*p* value
S1 -S0	4.245	0.313	13.552	<0.001
S2 - S0	6.530	0.316	20.632	<0.001
S3 - S0	4.980	0.442	11.27	<0.001
S2 - S1	2.285	0.349	6.554	<0.001
S3 - S1	0.735	0.505	1.456	0.473
S3 - S2	−1.550	0.442	3.505	0.006

Furthermore, simple effects analyses were conducted for the interaction effect (Bonferroni-corrected, α = 0.05) to elucidate the specific pattern of redness effects under different baseline sweetness levels (Table 4).

**Table 4:** Results of simple effects analyses (Bonferroni-corrected).

Baseline sweetness	Redness comparison	Mean difference (mm)	Standard error	*t* value	*p* value
Low	S1 - S0	5.457	0.632	8.638	<0.001
	S2 - S0	8.278	0.612	13.523	<0.001
	S3 - S0	5.997	0.987	6.077	<0.001
	S2 - S1	2.82	0.503	5.603	<0.001
	S3 - S1	0.54	1.012	0.533	0.95
	S3 - S2	−2.28	0.776	−2.939	0.027
Medium	S1 - S0	6.052	0.603	10.038	<0.001
	S2 - S0	9.867	0.676	14.604	<0.001
	S3 - S0	7.647	0.888	8.616	<0.001
	S2 - S1	3.815	0.823	4.637	<0.001
	S3 - S1	1.595	0.927	1.721	0.327
	S3 - S2	−2.22	0.876	−2.533	0.07
High	S1 - S0	1.225	0.595	2.058	0.185
	S2 - S0	1.445	0.583	2.476	0.08
	S3 - S0	1.295	0.652	1.988	0.21
	S2 - S1	0.22	0.421	0.523	0.953
	S3 - S1	0.07	0.692	0.101	1
	S3 - S2	−0.15	0.662	−0.227	0.996

Under the low-sweetness (L) condition, the redness effect was most pronounced. From S0 to S1, sweetness ratings increased significantly (5.457 mm, p < 0.001), indicating that even low redness effectively enhanced perceived sweetness. Ratings continued to increase significantly from S1 to S2 (2.82 mm, *p* < 0.001), reaching a peak. However, a significant decline was observed from S2 to S3 (−2.28 mm, p = 0.027). Nevertheless, the S3 ratings remained significantly higher than the S0 baseline (p < 0.001), indicating that although high redness partially weakened the enhancement effect, it did not completely eliminate it. This may be attributable to the negative cognition of “artificial colorant” elicited by the high concentration of carminic acid in some participants.

Under the medium-sweetness (M) condition, the redness effect remained significant but with reduced magnitude. The increase from S0 to S1 was 6.052 mm (p < 0.001), and from S1 to S2 was 3.815 mm (p < 0.01). Similarly, a slight decrease was observed from S2 to S3 (−2.22 mm, p = 0.07), and no significant difference was found between S3 and S1 (*p* = 0.327), although both were significantly higher than S0. These results indicate that cross-modal expectation continued to operate at this sweetness level; however, as the gustatory signal became sufficiently strong, the supplementary contribution of visual cues diminished. Moreover, the decline induced by high redness (S3) was significant under low sweetness (S2 vs. S3, *p* = 0.027) and marginally significant under medium sweetness (S2 vs. S3, *p* = 0.07), suggesting that higher baseline sweetness may partially buffer the negative interference associated with high redness.

Under the high-sweetness (H) condition, the redness effect was substantially compressed, and the enhancement effect of color approached saturation. Specifically, the increase from S0 to S1 was minimal (1.225 mm, *p* = 0.185), representing the smallest increment among the three sweetness levels; the increase from S1 to S2 further narrowed to 0.22 mm (*p* = 0.953), indicating a markedly attenuated marginal effect. No significant differences were observed among redness levels (all p > 0.05). This may indicate that under high-sweetness conditions, the main effect of red visual intensity largely disappeared at the behavioral level. The strong gustatory signal became dominant, while both the “artificial” impression and the “enhancement effect” of the visual cue were diluted by the baseline taste intensity, resulting in only weak random fluctuations in its modulatory effect.

Collectively, these findings robustly support the core hypotheses of the present study and provide refined behavioral evidence for visual–gustatory cross-modal integration, thereby establishing a clear behavioral phenotype to guide subsequent EEG analyses. Compared with the colorless solution (S0), solutions supplemented with carminic acid were perceived as significantly sweeter under most conditions. This effect was most pronounced under low sweetness, with a maximum relative increase of 26.48% (S2 condition), demonstrating that color is a powerful modulator of sweetness perception and supporting H1. Under low and medium sweetness levels, sweetness ratings consistently exhibited an inverted U-shaped nonlinear pattern with increasing redness. Medium redness (S2) was identified as the “optimal redness level” for maximal sweetness enhancement, whereas low redness (S1) can be considered the “initial enhancement threshold.” The systematic decline in sweetness ratings under the high-redness condition (S3) may be related to negative cognitive evaluations, such as a perceived “artificial” impression, thereby providing a cognitive-level explanation for the “inverted U-shaped” relationship and supporting H2. Furthermore, the enhancement effect of redness was significantly modulated by baseline sweetness, demonstrating clear intensity dependency. When the gustatory signal was weak (low sweetness), the supplementary gain effect of visual cues was most prominent. In contrast, under high sweetness conditions, the strong gustatory signal dominated perception, substantially diminishing the marginal gain of visual input and reflecting potential “cognitive resource competition” between sensory channels.

### 3.2 EEG analysis

After preprocessing the data from 1920 epochs (40 participants × 12 conditions × 4 repetitions) using the EEGLAB software, 231 trials (12%) were excluded due to artifact contamination (e.g., swallowing, eye movements, muscle activity) or signal drift. A total of 1689 valid trials were retained for subsequent analyses. To minimize systematic bias and account for inter-individual variability (Michel et al., 2025), resting-state baseline normalization was applied to the EEG data (Wang, 2024).

#### 3.2.1 EEG time-domain analysis

**Fig. 7:**
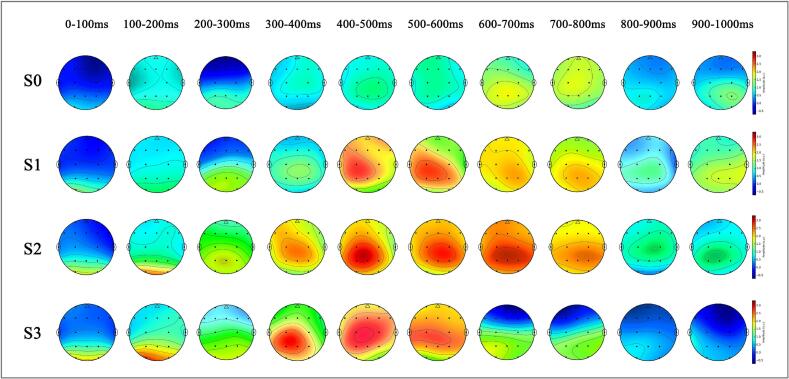
EEG topographic map sequences for medium-sweetness solutions under the four redness conditions (0–1000 ms). S0: colorless control; S1: low redness; S2: medium redness; S3: high redness.

To clearly illustrate how different redness levels influenced the neural processing of sweetness and its temporal dynamics, time-domain EEG responses elicited under different redness conditions were analyzed using the medium-sweetness solution (5.0% sucrose) as a representative condition. Fig. 7 presents the grand-average scalp topographies of all valid trials within the 0–1000 ms time window. The figure integrates EEG responses elicited by the four stimulus conditions (S0, S1, S2, S3) under the medium-sweetness solution (5.0% sucrose). Although this section focuses on the medium-sweetness condition, similar redness-modulated patterns—albeit with varying magnitudes—were also observed under low- and high-sweetness conditions, consistent with the intensity-dependent pattern identified in the behavioral results.

Overall, compared with the colorless control (S0), the red solution conditions (S1, S2, S3) elicited more positive-going EEG responses. However, this modulatory effect was not uniform across all participants, and its spatial topography was relatively complex. Under the colorless control condition (S0), EEG activity was relatively evenly distributed throughout the time window and of lower amplitude, with a peak around 600–700 ms, reflecting the baseline neural response to pure gustatory stimulation (sucrose).

After the introduction of a low concentration of carminic acid (S1, a* ≈ +10.0), the EEG activity pattern began to change. During the 200–300 ms time window, amplitude modulation of the negative wave of the N1–P2 complex was observed in the frontal and central regions (F(3,117) = 4.28, *p* = 0.007, η^2^ = 0.10), reflected as a relatively more positive shift. This may indicate that cross-modal expectations elicited by color cues modulated early sensory processing. The amplitude of the late positive component (LPC) during the later 400–600 ms time window showed an increasing trend, although the statistical strength was relatively weak. This enhancement of neural activity may be associated with the initial processing of color cues and the cross-modal expectation they evoked, which preset a cognitive expectation of “greater sweetness” for subsequent gustatory processing.

Under the medium redness condition (S2, a* ≈ +20.0), the most pronounced and consistent neural responses were elicited. Activation in the frontal–central regions became broader and stronger within the 300–700 ms window. In the parietal region (e.g., around electrode Pz), the LPC (400–600 ms) reached peak mean amplitude. This component is typically closely associated with attentional resource allocation and higher-order cognitive appraisal (Sun et al., 2024). In the context of the present study, it may reflect the brain's evaluative processing of the congruency between color-induced expectations and actual gustatory input. However, not all participants exhibited a canonical LPC pattern, and considerable inter-individual variability was observed.

When redness further increased to a high level (S3, a* ≈ +35.0), the neural response pattern became more differentiated. Although overall LPC amplitude remained higher than the S0 baseline, it was significantly reduced compared with S2 (*p* < 0.05), and its scalp distribution was more diffuse, without forming significant local clusters in statistical parametric mapping. Early frontal activation during the 200–400 ms time window was slightly weaker than that observed under S2, but the difference was not significant. During the later 400–800 ms time window, irregular and less synchronized activity emerged across widespread brain regions, including the temporal region. In some participants, sustained negative activity was observed in the frontal region, suggesting that this may be related to negative cognitive evaluations, such as a perceived “artificial” impression or mild discomfort elicited by the high concentration of carminic acid (E120). These additional cognitive processes may have interfered with and weakened effective sensory integration between color and taste, thereby accompanying the decline in sweetness perception observed at the behavioral level.

Repeated-measures ANOVA was performed on the mean amplitude within the 400–600 ms time window in the parietal ROI (Pz, P3, and P4). The results showed a significant main effect of redness (F (3, 117) = 12.47, *p* < 0.001, η^2^ = 0.25). FDR-corrected post hoc comparisons revealed that the amplitude under the S2 condition was significantly greater than that under the S0 (p < 0.001) and S3 (*p* = 0.013) conditions, whereas the differences between S1 and S0 (*p* > 0.05) and between S1 and S3 (p > 0.05) did not reach statistical significance. This response pattern was consistent with the behavioral “inverted U-shaped” relationship, providing corresponding correlative evidence at the level of neurophysiological activity for the behavioral findings.

#### 3.2.2 EEG frequency-domain analysis

**Fig. 8:**
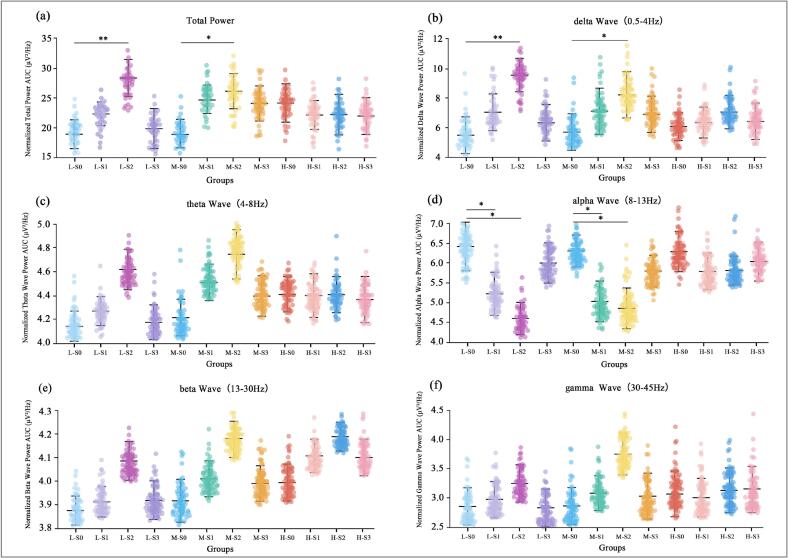
EEG rhythmic power changes induced by the 12 stimulus conditions. (a) Analysis of variance differences in whole-brain total power (0.5–45 Hz) across the 12 stimulus conditions. (b)–(f) Power differences in specific EEG rhythms (delta, theta, alpha, beta, and gamma bands) induced by the 12 stimuli, respectively. L: low sweetness; M: medium sweetness; H: high sweetness; S0: colorless control; S1: low redness; S2: medium redness; S3: high redness. Data are presented as mean ± standard deviation, with significance levels indicated as *P < 0.05 and **P < 0.01.
b. Delta band (0.5–4 Hz): Delta power changes are closely associated with emotional valence and reward processing. A 3 (sweetness) × 4 (redness) ANOVA conducted on delta-band power showed a significant main effect of redness (F(3,117) = 4.38, *p* = 0.006, η^2^ = 0.11). Compared with S0 and S1, the S2 condition induced enhanced delta power (event-related synchronization, ERS; *p* < 0.01), particularly in frontal and central regions. This effect was most pronounced under low sweetness and may reflect increased hedonic valuation of sweetness induced by red visual cues.
c. Theta band (4–8 Hz): Frontal theta power showed a modest increase under the S2 condition; however, this effect did not remain significant after correction for multiple comparisons (F(3,117) = 2.89, *p* = 0.038, η^2^ = 0.07; p-FDR = 0.062).
d. Alpha band (8–13 Hz): Alpha activity reflects attentional allocation and cognitive control. The modulatory effect of redness on alpha-band event-related desynchronization (ERD) was significant (F(3,117) = 6.71, p < 0.001, η^2^ = 0.15). This ERD effect was most pronounced in the frontal and parietal regions, and its magnitude was significantly stronger under the S1 and S2 conditions than under the S0 condition (*p* < 0.05). These findings indicate that visual cues with low to medium redness may effectively recruit cortical attentional resources. Under the high-redness condition (S3), alpha ERD remained present but became spatially diffuse, suggesting disrupted cognitive processing, consistent with the behavioral attenuation of sweetness enhancement.
e. Beta band (13–30 Hz): Beta oscillations are associated with cognitive evaluation and sensorimotor integration. Across conditions, beta-band activity did not show a global significant difference (F(3,117) = 1.23, *p* = 0.301, η^2^ = 0.03), with only nonsignificant power fluctuations observed during the late stimulus period (600–900 ms), which may be related to motor processes such as preparation for expectoration.
f. Gamma band (30–45 Hz): As an exploratory index of cross-modal integration, gamma-band activity showed a weak trend for the main effect of redness at the whole-brain level (F(3,117) = 2.95, *p* = 0.035, η^2^ = 0.07). However, this effect did not survive FDR correction for multiple frequency comparisons (p-FDR = 0.082). This may be because gamma-band signals are susceptible to contamination by artifacts such as electromyographic activity, and the responses showed substantial inter-individual variability, with only approximately 25% of participants exhibiting clear event-related synchronization. Therefore, the present study does not draw any cognitive mechanistic inferences from this exploratory finding, but instead regards it as a potential electrophysiological phenotype that requires further validation in future studies using techniques with greater resistance to interference, such as magnetoencephalography (MEG) or intracranial electrodes.

A 3 (sweetness: L, M, H) × 4 (redness: S0, S1, S2, S3) repeated-measures ANOVA was conducted on whole-brain total power (0.5–45 Hz). The results showed a significant main effect of sweetness (F(2,78) = 8.93, p < 0.001, η^2^ = 0.19), a significant main effect of redness (F(3,117) = 15.62, p < 0.001, η^2^ = 0.30), and a significant interaction between the two factors (F(6,222) = 3.45, *p* = 0.003, η^2^ = 0.09). These findings indicate that different redness levels elicited significant differences in overall EEG activity and that these differences were modulated by baseline sweetness.

a. To delineate the specific contributions of different frequency bands, the same two-factor ANOVA was conducted for each oscillatory band. The patterns of power differences across conditions are summarized in Fig. 8. Overall, the robustness of frequency-domain effects was lower than that observed in time-domain analyses, with several bands showing relatively small effect sizes (η^2^ < 0.10), reflecting the high variability inherent in EEG oscillatory signals.

#### 3.2.3 EEG dynamic time–frequency analysis results

**Fig. 9:**
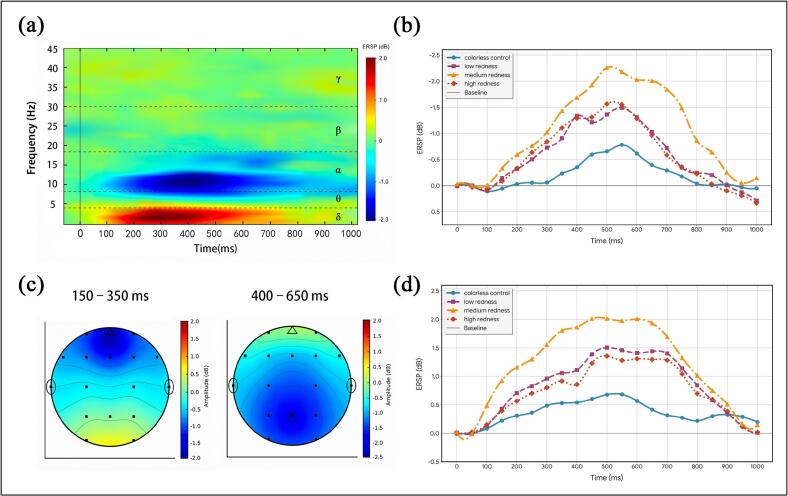
(a) Full-band ERSP heatmap of the parietal ROI under the medium-redness (S2) condition (low sweetness). (b) Alpha-band ERSP time-course curves for the four redness levels under the low-sweetness condition (parietal ROI, *n* = 40). (c) Topographic map of the Alpha ERD difference between the S2 and S0 conditions. (d) Delta-band ERSP time-course curves for the four redness levels under the low-sweetness condition (frontal Fz, n = 40).

To reveal the temporal dynamics of the modulatory effects across different frequency bands, ERSP was further used to perform dynamic time–frequency decomposition of the EEG data. Fig. 9a presents the full-band ERSP heatmap of the parietal ROI under the medium-redness (S2) × low-sweetness condition as a representative example.

In the alpha band (8–13 Hz), dynamic time–frequency analysis revealed a relatively clear two-stage spatial and temporal distribution pattern (Fig. 9b and c). In the first stage (150–350 ms), all colored conditions showed an initial alpha ERD. Because the visual cues had been presented before the gustatory stimulus, this early frontal ERD may reflect rapid cross-modal matching and initial integration between prior visual expectations and actual gustatory input. The early frontal ERD induced by medium redness (S2) showed the greatest magnitude (−1.7 dB); high redness (S3) was slightly weaker than S2 but did not differ significantly (difference < 0.3 dB, *p* > 0.10), indicating that high redness was not impaired during the early stages of feature extraction and sensory gating. In the second stage (350–700 ms), alpha ERD subsequently extended from the frontal region to the parietal region (Pz, P3, and P4), forming a peak fronto-parietal distribution at 400–550 ms (Fig. 9b). The parietal ERD peak was strongest under the S2 condition (−2.3 dB). In contrast, high redness (S3) exhibited a “biphasic dissociation” pattern: its early frontal ERD (150–350 ms) did not differ significantly from that under S2 (p > 0.10), whereas its late parietal ERD (400–650 ms) was significantly weaker than that under S2 (*p* = 0.04). This temporal dissociation suggests that the interference of high redness with cross-modal integration may originate from the late cognitive appraisal stage of integration.

Analysis of the delta band (0.5–4 Hz) showed (Fig. 9d) that, under colored conditions, broad synchronization enhancement was observed in the frontal midline region (Fz) during the early-to-middle time window after gustatory stimulation, approximately 200–800 ms, with the energy core mainly concentrated around 500 ms (S2 condition, low sweetness: +1.9 dB). The onset timing of this delta ERS showed a synchronous trend with frontal alpha ERD, suggesting temporal neural coordination between reward expectation activation and attentional anticipatory preparation.

Apart from the significant effects described above, the remaining frequency bands (theta, beta, and gamma) did not form significant time–frequency clusters after cluster correction (cluster-corrected, *p* > 0.05).

### 3.3 Correlation analysis between EEG indices and subjective sweetness ratings

**Fig. 10:**
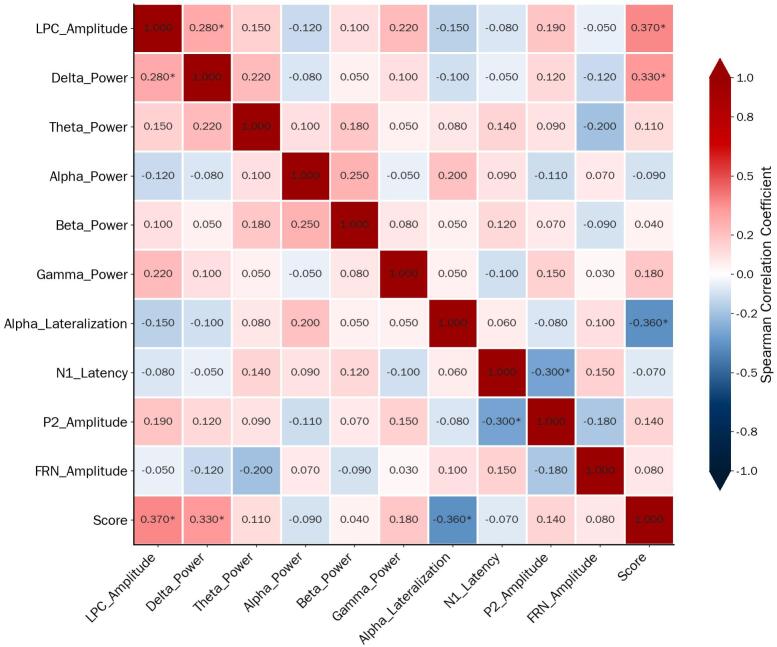
Spearman correlation matrix between EEG indices and behavioral sweetness ratings (Significance levels are indicated as *p < 0.05).

To examine the association between neural activity and behavioral perception (Hypothesis H3), Spearman correlation analyses were conducted between key EEG indices and subjective sweetness intensity ratings (VAS) across all experimental conditions (3 sweetness × 4 redness) for the 40 participants. The correlation matrix between the principal EEG indices and subjective sweetness ratings is presented in Fig. 10.

The analysis revealed a moderate positive correlation between the mean parietal LPC amplitude (400–600 ms) and subjective sweetness ratings (*r* = 0.37, p < 0.05). After excluding two participants identified as outliers, the correlation increased to *r* = 0.41. This suggests that changes in parietal LPC amplitude may occur in coordination with changes in subjective sweetness intensity. One possible explanation is that a stronger sweetness experience can capture more attentional resources and elicit deeper cognitive appraisal, thereby manifesting as an enhancement of LPC amplitude.

The overall correlation between gamma-band event-related synchronization (ERS) power in the fronto-parietal network during the 300–600 ms time window and subjective sweetness ratings did not reach statistical significance (*r* = 0.18, *p* = 0.098). Meanwhile, delta-band (0.5–4 Hz) power, which is associated with pleasantness and reward processing, was also positively correlated with sweetness ratings (*r* = 0.33, p < 0.05). This provides supportive neurophysiological evidence that a sweeter perceptual experience is accompanied by greater hedonic value.

Notably, the alpha power lateralization index (left/right frontal alpha power ratio) showed a significant negative correlation with subjective sweetness ratings (*r* = −0.36, p < 0.05). Higher alpha power generally reflects cortical inhibition (De Pesters et al., 2016), and its lateralization has been linked to affective valence processing (Wager et al., 2003). This negative correlation indicates that more balanced frontal activity may predict a more positive sweetness experience.

In summary, the positive correlation between parietal LPC amplitude and subjective sweetness ratings supports the potential of EEG as an objective evaluation tool in sensory science. Meanwhile, alpha-band lateralization may also serve as a complementary neural index. These objective neurophysiological findings not only support hypothesis H3, but also indicate that EEG can capture quantifiable brain activity signals that covary with subjective perception. This provides preliminary scientific evidence for using neural indices to objectively evaluate cross-modal sensory effects and to assist and complement subjective reports, with important practical value for optimizing color formulation strategies in the food industry under the context of sugar reduction.

## 4 Discussion

This study systematically investigated the cross-modal enhancement effect of red visual cues on sweetness perception and its underlying neural dynamics by integrating behavioral measures with EEG techniques. At the behavioral level, the enhancement effect of red color on sweetness perception followed an inverted U-shaped non-linear relationship and exhibited clear intensity dependence. At the neural level, the late positive component (LPC) over parietal regions was identified as a key electrophysiological marker of this enhancement effect, with its amplitude showing a significant correlation with subjective ratings. The study confirms the behavioral efficacy of color as a sensory modulation tool and provides neural evidence for the underlying complex neural mechanisms.

### 4.1 Nonlinear effects of red visual cues on sweetness perception

By demonstrating that the sweetness-enhancing effect of redness follows a nonlinear inverted U-shaped relationship, the present study provides empirical support for the classical “color–taste” cross-modal correspondence theory (Spence, 2011, Spence, 2015). The use of a finely graded redness manipulation overcomes the limitations of earlier studies that relied primarily on binary comparisons such as “presence/absence” or “high/low” color intensity (Johnson & Clydesdale, 1982; Maga, 1974), thereby offering direct evidence for a dose–response relationship in cross-modal integration.

From a cognitive perspective, the ascending phase of the inverted U-shaped curve (S0 to S2) can be explained by cross-modal expectation theory (Koch & Koch, 2003; Peng et al., 2022). Red, which has been strongly associated with “sweetness” and “ripeness” through long-term dietary experience (Saluja & Stevenson, 2018), may activate implicit “red–sweet” associations in memory and modulate gustatory processing via top-down mechanisms. Medium redness (S2) appears to represent the “optimal gain point” because it effectively triggers positive expectations without exceeding the perceptual threshold of naturalness. This finding aligns closely with Spence's (2022) concept of “cue validity boundaries,” which posits that visual cues exert maximal modulatory effects within an optimal range—too weak to activate associations when insufficient, and prone to cognitive conflict when excessive.

The descending phase of the curve (S2 to S3) may reflect a trade-off in cognitive evaluation. The decline in perceived sweetness under high redness (S3) is unlikely to represent a simple saturation effect; rather, it may result from the intrusion of perceived artificiality. When redness exceeds the typical range encountered in natural foods, participants may engage prefrontal cognitive control processes (Sugrue & Dando, 2018), reappraising the color cue from a “flavor expectation signal” to an “artificial additive.” Such negative expectations may partially counteract the positive “red–sweet” association. In addition, the increased dispersion of ratings induced by high redness under the low-sweetness condition may reflect inter-individual differences in participants' cognitive appraisal of the perceived “artificial” impression. Specifically, some participants may have remained dominated by the color cue, whereas others may have engaged cognitive control mechanisms, thereby weakening the color-induced enhancement effect.

This interpretation received preliminary support from the EEG data: enhanced late frontal negative activity and reduced LPC amplitude were observed under the S3 condition, which is consistent with the expected resource dispersion induced by cognitive conflict (Sun et al., 2024; Wu et al., 2023). Notably, even under the S3 condition, sweetness ratings remained higher than the S0 baseline, suggesting that positive associations and potential trade-offs related to perceived artificiality may occur in parallel rather than being mutually exclusive. This finding provides a complementary perspective to the single-pathway theory proposed by Spence. Nevertheless, because the present study was originally designed to quantify sweetness intensity and did not include direct indicators such as hedonic ratings or perceived naturalness, the conclusion that the perceptual decline was driven by perceived artificiality remains somewhat speculative. Future studies should further clarify the psychological mechanisms underlying this perceptual decline by incorporating multidimensional sensory evaluation scales.

Compared with early investigations (Alley & Alley, 1998; Maga, 1974), the present study not only confirms the sweetness-enhancing effect of color but also quantitatively links physical color properties to perceptual outcomes through controlled manipulation of CIE a* values. By identifying both an “optimal sweetness-enhancing redness” and an “initial enhancement threshold,” the study responds directly to the call by Motoki et al. (2023) for more precise quantification of sensory modulation effects. Future research on color–taste interactions should further strengthen dose–response modeling between physical attributes and perceptual effects to enhance translational and practical value in food formulation science.

### 4.2 Intensity dependence and resource competition in multisensory integration

The quantitative results of the present study indicate that the absolute magnitude of color-induced enhancement was greatest under medium sweetness, whereas the relative enhancement rate was highest under low sweetness. Under high sweetness, however, as the gustatory signal approached saturation, the modulatory effect of color was markedly compressed, demonstrating clear intensity dependency. This mechanism can be interpreted through two complementary theoretical frameworks. First, Bruno's “sensory weighting model” proposes that the brain assigns weights to inputs from different sensory channels according to their relative reliability; when the gustatory signal (high sweetness) is sufficiently strong, the perceptual weight assigned to visual cues is substantially reduced (Bruno, 2018). Second, Wu et al.'s (2023) “cognitive resource competition theory” suggests that high-intensity gustatory input occupies substantial attentional resources, thereby diminishing the depth of processing allocated to visual cues.

The EEG findings provide direct neural evidence supporting this mechanism. Under low sweetness, alpha desynchronization (reflecting increased attentional engagement) was significantly enhanced, and delta power (associated with hedonic processing) was elevated, indicating that visual cues were deeply integrated into gustatory perception. In contrast, under high sweetness, these neural effects were attenuated. LPC amplitude was reduced and its scalp distribution became more diffuse, suggesting decreased resource allocation for cross-modal integration. These findings are highly consistent with Zhang et al. (2023), who reported stronger aroma-induced sweetness enhancement under weak gustatory signals in aroma–taste interaction studies. Together, the evidence suggests that intensity dependency may represent a general principle of cross-modal sensory interaction rather than a phenomenon specific to color–taste correspondence.

From a theoretical perspective, the present findings extend the traditionally static view of cross-modal correspondence theory (Spence, 2011) by demonstrating that color–taste associations are dynamically modulated by contextual sensory intensity. Red color does not enhance sweetness under all conditions; rather, it exerts its greatest effect when the gustatory signal is relatively weak, as in low-sugar products. This finding provides important practical guidance for the food industry. In light of the World Health Organization (WHO), 2015 sugar reduction guidelines and Peñalvo's (2024) research on sugar tax policies, color optimization strategies should be preferentially applied to low-sugar and sugar-free products to precisely compensate for sweetness loss, whereas excessive reliance on colorants in high-sugar products would be inconsistent with the goal of developing healthier formulations. In addition, this color-induced enhancement effect could be integrated with a “stepwise sugar reduction” process, using visual gain to buffer the flavor gap during the initial stages of sugar concentration reduction.

Moreover, by employing a systematic sweetness gradient design, the present study delineates the effective range of color-induced enhancement (low to medium sweetness), providing a standardized reference for future experimental design. Previous research has often assumed that red universally signals “greater sweetness” (Paakki, Sandell and Hopia, 2019). However, the current findings demonstrate that under high baseline sweetness, due to sensory saturation and gustatory dominance, the enhancement effect of color becomes negligible. This finding suggests that, in practical food product development, the baseline sensory characteristics of the product must be considered to avoid overgeneralization.

### 4.3 Neural temporal dynamics of cross-modal integration

At the neural level, although fMRI studies have identified the orbitofrontal cortex (OFC) and anterior insula as key regions in flavor perception (Avery et al., 2020; Morales & Berridge, 2020), the present study leverages the high temporal resolution of EEG to provide evidence regarding the time course of activity in higher-order integrative processes.

In the early sensory initiation stage (200–300 ms), a frontal N1–P2 complex modulated by different redness levels was observed. In cross-channel attention studies, this early potential change is commonly interpreted as reflecting sensory gating and attentional orienting to task-relevant multimodal input signals (Favero et al., 2023). This may suggest that, during the early stage of sensory processing, red visual cues rapidly adjusted the preparatory state of cortical responses to subsequent gustatory signals through top-down attentional mechanisms. This finding is consistent with the EEG results reported by Domracheva et al. (2020), demonstrating that visual information begins to modulate gustatory processing at early perceptual stages, thereby laying the foundation for subsequent integration.

In the intermediate cross-modal matching stage (400–600 ms), parietal LPC amplitude reached its peak and was significantly positively correlated with subjective sweetness ratings. In the present study, the LPC was systematically modulated by the experimental conditions. Given its well-established role as an index of attention and cognitive appraisal (Mheich et al., 2021), its amplitude reached a peak under the medium-redness condition (S2) and covaried with subjective perceptual intensity. This enhancement may indicate that when medium redness (S2) was highly congruent with gustatory input, such sensory congruency attracted more cognitive resources for in-depth evaluation, thereby manifesting as an increased LPC amplitude. This is consistent with the theory proposed by Small and Prescott (2005) , which suggests that the OFC serves as a hub for cross-modal integration. In this context, the LPC may reflect cognitive appraisal activity in higher-order cortical regions, such as the parietal cortex, after receiving signals derived from multisensory integration.

During the late cognitive appraisal stage (600–800 ms), enhanced frontal negative activity was observed under the high-redness condition (S3). This neural activity pattern is consistent with the trend reported in studies on cognitive conflict potentially induced by a perceived artificial impression (Khan et al., 2025). Although some studies have associated gamma-band oscillations with cross-modal feature binding (Buzsáki and Wang, 2012), the validity of the gamma band as a general indicator of cross-modal integration remains to be further confirmed in the present study because of insufficient statistical significance.

Frequency-domain analyses provide a complementary perspective on the oscillatory mechanisms underlying cross-modal integration. Previous studies have demonstrated that frontal alpha asymmetry is closely associated with affective valence processing, with left-dominant activity typically linked to approach motivation and positive affect. The present study found that the lateralization index of alpha-band power was significantly negatively correlated with subjective sweetness ratings, which may reflect the asymmetry of frontal emotional processing: more balanced alpha activity may be associated with a more positive sweetness experience. This suggests that the enhancement effect of color involves not only sensory integration, but may also modulate the hedonic value of flavor through emotional pathways. However, given the limited source-localization capability of EEG, the activation patterns reported in this study reflect scalp-level potential changes rather than direct measurements of activity in specific deep brain regions. Future studies should therefore combine techniques with higher spatial resolution and high-density EEG to verify the extent to which these scalp EEG-based oscillatory activities represent the true functional states of specific brain networks.

**Fig. 11:**
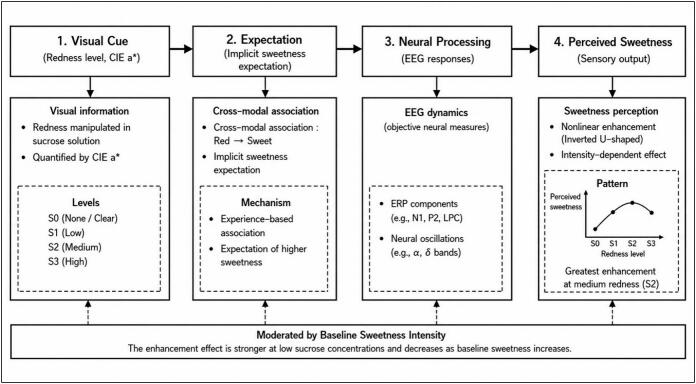
Conceptual model of the cognitive and neural mechanisms underlying the modulation of sweetness perception by redness.

Theoretically, the present findings integrate “top-down expectation modulation” (Koch & Koch, 2003) with “bottom-up sensory integration” frameworks (Stein et al., 2014). In the early stage, the process was mainly dominated by top-down expectation priming; in the middle stage, cognitive resources were allocated to sensory matching and evaluation; and in the late stage, cognitive appraisal may have modulated the integration outcome. These findings indicate that the redness-induced enhancement of sweetness may be more closely associated with the late cognitive appraisal stage of perceptual processing, rather than purely with early sensory gating. This supports the notion that flavor perception is actively constructed by the brain rather than passively received (Stern, 2020). Color, as a priori visual context, exerts top-down modulation on the cortical construction of gustatory experience. In doing so, the findings extend the neural model of food reward proposed by De Araujo et al., (2020) by incorporating visual cues into the neural circuitry of flavor perception and clarifying their modulatory role in gustatory reward processing, as evidenced by the positive association between delta power and sweetness ratings. The above three-stage neural processing sequence and its theoretical integration together constitute a preliminary conceptual model of the cognitive and neural mechanisms by which redness modulates sweetness perception (Fig. 11).

At the application level, the neural indices identified in this study, including the LPC, delta-band activity, and alpha-band lateralization, provide potential objective neurophysiological evidence for food sensory evaluation and formulation design, serving as a complement to subjective reports. To some extent, these indicators may help address the limitations of traditional subjective ratings, such as large inter-individual variability and susceptibility to bias (Shariati et al., 2025). These indices could be used during early-stage food product development to rapidly and sensitively detect consumers' implicit neural responses to cross-modal formulations, thereby facilitating the screening of potential optimization strategies.

### 4.4 Individual differences analysis

By calculating the individual color enhancement magnitude (S2 − S0), two distinct perceptual profiles were identified: visually dependent (82.5%, *n* = 33) and non-visually dependent (17.5%, *n* = 7). Visually dependent participants exhibited a mean S2 − S0 increase of 9.64 mm under low sweetness and 9.41 mm under medium sweetness, indicating a strong “red–sweet” cross-modal association and a greater tendency to allocate attentional resources to visual cues during gustatory processing. In contrast, non-visually dependent participants showed a markedly smaller enhancement under low sweetness (mean increase = 1.86 mm), and their subjective reports frequently emphasized reliance on “taste alone.” However, given the relatively small sample size of the latter subgroup, meaningful statistical comparisons were not feasible, limiting the generalizability of these conclusions. Nonetheless, the findings provide preliminary evidence that the efficiency of cross-modal integration is not uniform but may reflect the combined influence of genetic traits, cognitive style, and prior dietary experience. Holgerson et al. (2023) reported that genetic variation in sugar preference is associated with polymorphisms in the T1R2/T1R3 receptor genes. It is possible that visually dependent participants possess stronger genetically mediated “red–sweet” associative tendencies or lower gustatory receptor sensitivity, thereby relying more heavily on visual cues for perceptual supplementation. Peng et al. (2022) further demonstrated that individuals with a “visual-dominant” cognitive style are more susceptible to color cues, whereas non-visually dependent participants in the present study may exhibit a “gustatory-dominant” cognitive style, placing greater emphasis on intrinsic taste attributes. Moreover, habitual consumption of “natural” or uncolored foods may attenuate learned color–taste associations (Saluja & Stevenson, 2018), thereby reducing the magnitude of color-induced enhancement effects.

This finding on inter-individual differences has important practical implications for developing personalized strategies for sugar reduction and low-sugar food formulation optimization. In conjunction with Liu et al.'s (2022) research on the neural basis of sugar preference, future studies could identify consumers' perceptual phenotypes through behavioral testing. For visually dependent consumers, medium redness may serve as an effective approach to compensating for sweetness perception in low-sugar beverages. For non-dependent consumers, however, synergistic interventions involving other sensory channels, such as olfaction (Oliveira et al., 2021) and texture, may be required.

Notably, visually dependent participants accounted for 82.5% of the sample, suggesting that although inter-individual variability exists, the majority of consumers are sensitive to the sweetness-enhancing effect of red coloration. This provides a population-level basis for the practical implementation of color-based strategies. Future studies with larger samples, incorporating genome-wide association analyses (GWAS) and detailed dietary history assessments, may further clarify the genetic and environmental determinants of individual differences in color–taste integration.

### 4.5 Research limitations and translation into food formulation

The ecological validity of the present study has several limitations. First, to strictly control confounding variables and extract relatively pure EEG neural signals associated with color–taste cross-modal processing, the study employed a highly simplified experimental stimulus system consisting only of purified water, sucrose, and a single colorant, carminic acid. However, real food and beverage systems, such as fruit juices and dairy products, are highly complex physicochemical food matrices. Therefore, in the absence of complex beverage matrices, the “optimal sweetness-enhancing redness” proposed in this study (S2, a* ≈ +20.0) should be regarded as a perceptual and neural response pattern observed under controlled conditions. This optimal value is likely to shift with changes in flavor background, formulation structure, and flavor–color congruency. When translated into practical formulation applications, the effectiveness of color–taste integration will inevitably be influenced by the interactions of various real-matrix factors, such as volatile aroma compounds, acidity, texture, and ingredient interactions.

Therefore, future research should extend the experimental stimuli from simple sucrose solutions to complex real food systems. Subsequent studies are recommended to adopt multivariate orthogonal designs to systematically quantify the interaction weights of multisensory dimensions, including color, volatile aroma, pH, and viscosity, in reshaping the dose–response curve of “optimal redness.” On this basis, the generalizability of related EEG neural indices, such as LPC amplitude and alpha-band lateralization, should be further validated, thereby providing formulation guidelines with greater translational value for the development of low-sugar foods.

Second, although EEG has millisecond-level temporal resolution, its spatial resolution is limited. The parietal LPC activity observed in this study may originate from a precise cortical network involving multiple brain regions, such as the OFC and insula, but EEG cannot accurately localize these sources. Future research should combine the spatial resolution advantages of fMRI with the temporal resolution advantages of EEG to clarify the precise neural sources of indices such as the LPC and deepen the understanding of the underlying neural mechanisms.

Finally, in terms of evaluation dimensions, the present study mainly focused on the dose–response relationship between redness and sweetness intensity, but did not directly measure participants' emotional experiences. Future studies could incorporate real-time ratings of sample pleasantness (hedonic ratings) and perceived naturalness, and use mediation analysis to quantitatively verify whether redness modulates the effectiveness of cross-modal sweetness enhancement by altering the perceived natural attributes of the product. In addition, the participants in this study were healthy young adults tested under strictly controlled laboratory conditions, resulting in a relatively homogeneous sample. Therefore, the generalizability of the findings to broader age groups, cultural backgrounds, or real-world consumption contexts remains to be further explored. Future research should include participants with different ages, cultural backgrounds, and metabolic states to examine both the generality and specificity of inter-individual differences.

## 5 Conclusion

This study advances research on color–taste interactions from descriptive behavioral associations to mechanistic explanation. By introducing CIE a*-quantified carminic acid redness as a controlled visual variable, the present work demonstrates a nonlinear and intensity-dependent modulation of sweetness perception, clarifies the dose–response relationship between color intensity and perceptual enhancement, and integrates two core cognitive mechanisms: cognitive resource competition and artificiality-related trade-off. Behavioral results clearly confirmed that, compared with colorless solutions, sucrose solutions with added carminic acid colorant were perceived as significantly sweeter (supporting H1), highlighting the substantial sensory compensation potential of visual color cues as a controllable formulation parameter in low-sugar and sugar-reduced products. Moving beyond binary comparisons of “presence/absence” or “high/low” chromaticity, this study quantitatively linked physical color attributes to perceptual outcomes and revealed a stable inverted U-shaped nonlinear relationship between redness intensity and perceived sweetness (supporting H2). Medium redness (S2, a* ≈ +20.0) was identified as the “optimal sweetness-enhancing redness,” whereas low redness (S1, a* ≈ +10.0) represented the “initial enhancement threshold,” thereby defining the effective operating range and boundary conditions for redness as a tunable formulation variable. At the neural level, key EEG markers covarying with subjective sweetness enhancement were identified. Specifically, the parietal Late Positive Component (LPC) reached its peak under the medium-redness (S2) condition and showed a significant positive correlation with subjective sweetness ratings (supporting H3).

Through the systematic integration of behavioral and EEG approaches, the present study reveals a nonlinear, intensity-dependent cross-modal enhancement effect of red visual cues on sweetness perception and provides multi-stage neural evidence supporting this mechanism. The quantitative indices proposed in this study, including the “optimal sweetness-enhancing redness” (medium redness, a* ≈ +20.0) and the “intensity-dependent range” (low to medium sweetness, 2.5%–5.0% sucrose), provide preliminary and scientifically grounded reference points for sugar-reduction formulation design in the food industry. The neural activity patterns associated with cross-modal integration identified in this study, including the LPC, the temporal evolution of alpha ERD, delta-band activity, and alpha-band lateralization, further complement the research framework of sensory science by supporting its transition from subjective evaluation toward objective quantification. In the future, with the expansion of research targets, the integration of sensory quantification technologies, and the assessment of long-term effects, food color, as a key cue in multisensory integration, is expected to play a greater role in promoting healthier dietary behaviors and addressing the global challenges of obesity and diabetes control.

## CRediT authorship contribution statement

**Yifei Sun:** Writing – original draft, Visualization, Formal analysis, Data curation. **Yihan Wang:** Validation, Funding acquisition, Conceptualization. **Yang Li:** Methodology, Investigation, Formal analysis. **Feijie Xia:** Visualization, Validation, Conceptualization. **Tieyu Qian:** Supervision, Project administration, Conceptualization. **Yangyang Wei:** Writing – review & editing, Resources, Formal analysis, Conceptualization.

## Declaration of competing interest

The authors declare that they have no known competing financial interests or personal relationships that could have appeared to influence the work reported in this paper.

## Data Availability

Data will be made available on request.
