# Supplementary material for: Nonlinear and intensity-dependent enhancement of sweetness perception as a function of redness: Behavioral and EEG evidence for visual–gustatory cross-modal integration
Source: Food Chem X. 2026 Jun 6;37:104075. doi: 10.1016/j.fochx.2026.104075 (PMC13276578; doi:10.1016/j.fochx.2026.104075)
Supplement: Supplementary file 1 — Supplementary material [file mmc1.docx]

## **Supplementary material**

## **Table S1. CIE L*a*b* color coordinates of sucrose solutions with different red color levels**

Values are expressed as mean ± SD (n = 9; three independent preparations × three repeated measurements). Measurements were performed under D65 illumination using a spectrophotometer (Konica Minolta CM-5).

| **Color level** | **L* (Lightness)** | **a* (Red–Green)** | **b* (Yellow–Blue)** | $\mathbf{C}_{\mathbf{ab}}^{\mathbf{*}}$  **(Chroma)** | **h***  **(Hue angle)** |
| --- | --- | --- | --- | --- | --- |
| **S0**  (colorless) | 97.42 ± 0.38 | −0.92 ± 0.21 | 0.34 ± 0.27 | 0.99 ± 0.24 | 159.6 ± 12.8 |
| **S1**  (low redness) | 95.31 ± 0.44 | 9.84 ± 0.63 | 1.12 ± 0.41 | 9.90 ± 0.66 | 6.5 ± 2.4 |
| **S2**  (medium redness) | 92.07 ± 0.51 | 20.36 ± 0.78 | 1.48 ± 0.53 | 20.41 ± 0.80 | 4.2 ± 1.9 |
| **S3**  (high redness) | 87.26 ± 0.69 | 34.91 ± 1.12 | 2.06 ± 0.74 | 35.00 ± 1.14 | 3.4 ± 1.6 |

As shown in Table S1, increasing red color level resulted in a monotonic increase in a* and $C_{\mathrm{ab}}^{*}$, accompanied by a moderate decrease in L*. Hue angle remained relatively constant across color levels, indicating that color manipulation was primarily confined to the redness dimension rather than involving hue shifts.
